# Preliminary insights into the genetics of bank vole tolerance to Puumala hantavirus in Sweden

**DOI:** 10.1002/ece3.4603

**Published:** 2018-10-26

**Authors:** Audrey Rohfritsch, Maxime Galan, Mathieu Gautier, Karim Gharbi, Gert Olsson, Bernhard Gschloessl, Caroline Zeimes, Sophie VanWambeke, Renaud Vitalis, Nathalie Charbonnel

**Affiliations:** ^1^ CBGP, INRA, CIRAD, IRD, Montpellier SupAgro Univ. Montpellier Montpellier France; ^2^ Norwich Research Park Earlham Institute Norwich, Norfolk UK; ^3^ Department of Wildlife, Fish, and Environmental Studies SLU Umeå Sweden; ^4^ Georges Lemaître Centre for Earth and Climate Research, Earth and Life Institute Université Catholique de Louvain (UCL) Louvain‐la‐Neuve Belgium

**Keywords:** adaptation, hantavirus, immunity, molecular epidemiology, RAD sequencing, selection, tolerance, voles

## Abstract

Natural reservoirs of zoonotic pathogens generally seem to be capable of tolerating infections. Tolerance and its underlying mechanisms remain difficult to assess using experiments or wildlife surveys. High‐throughput sequencing technologies give the opportunity to investigate the genetic bases of tolerance, and the variability of its mechanisms in natural populations. In particular, population genomics may provide preliminary insights into the genes shaping tolerance and potentially influencing epidemiological dynamics. Here, we addressed these questions in the bank vole *Myodes glareolus*, the specific asymptomatic reservoir host of Puumala hantavirus (PUUV), which causes nephropathia epidemica (NE) in humans. Despite the continuous spatial distribution of *M. glareolus* in Sweden, NE is endemic to the northern part of the country. Northern bank vole populations in Sweden might exhibit tolerance strategies as a result of coadaptation with PUUV. This may favor the circulation and maintenance of PUUV and lead to high spatial risk of NE in northern Sweden. We performed a genome‐scan study to detect signatures of selection potentially correlated with spatial variations in tolerance to PUUV. We analyzed six bank vole populations from Sweden, sampled from northern NE‐endemic to southern NE‐free areas. We combined candidate gene analyses (*Tlr4*, *Tlr7*, and *Mx2* genes) and high‐throughput sequencing of restriction site‐associated DNA (RAD) markers. Outlier loci showed high levels of genetic differentiation and significant associations with environmental data including variations in the regional number of NE human cases. Among the 108 outliers that matched to mouse protein‐coding genes, 14 corresponded to immune‐related genes. The main biological pathways found to be significantly enriched corresponded to immune processes and responses to hantavirus, including the regulation of cytokine productions, TLR cascades, and IL‐7, VEGF, and JAK–STAT signaling. In the future, genome‐scan replicates and functional experimentations should enable to assess the role of these biological pathways in *M. glareolus* tolerance to PUUV.

## Graphical abstract

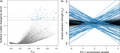

## 1 INTRODUCTION

The emergence of infectious pathogens of wild animal origin has become a significant health issue these last decades (Cunningham, Daszak, & Wood, 2017; Jones et al., 2008). In most cases, zoonotic agents are non pathogenic to their reservoir hosts: They are maintained in the long term without causing any clinical or behavioral symptoms to the latter (Haydon, Cleaveland, Taylor, & Laurenson, 2002). Characterizing such tolerance strategies and identifying the underlying biological processes at play still remain difficult both experimentally and through natural population surveys (see for details Mandl et al., 2015). The recent advent of high‐throughput sequencing technologies has shed some light on the genetic bases of the reservoir ability to tolerate infections, with some studies stressing the importance of immune‐related genes (e.g., Mandl et al., 2015, Raberg, Sim, & Read, 2007, Howick & Lazzaro, 2017). In particular, comparative genomics of reservoir and non reservoir species contributed to the characterization of specific candidate genes involved in tolerance (Mandl et al., 2015). Furthermore, transcriptomic analyses allowed the identification of some genes, presumably involved in tolerance, whose expression profiles before and after infection differ in reservoir and non reservoir host species (Smith et al., 2015). Although less documented, the variation of tolerance response within species is genuine (Hayward et al., 2014; Jackson et al., 2014) and influences the risk of zoonotic disease emergence. At such microevolutionary scale, population genomics is a valuable approach to characterize the correlations between genetic polymorphisms and variations in tolerance, a necessary step to decipher the eco‐evolutionary processes shaping these polymorphisms in natural populations.

The bank vole, *Myodes glareolus*, is the specific reservoir host of the hantavirus Puumala (PUUV), the causative agent of nephropathia epidemica (NE) in humans (Brummer‐Korvenkotio, Henttonen, & Vaheri, 1982). In *M. glareolus*, PUUV infections are chronic (Hardestam et al., 2008; Voutilainen et al.., 2015) and mainly asymptomatic (Bernshtein et al., 1999), although small negative effects on the fitness of the host have been reported (Kallio et al., 2007; Kallio, Helle, Koskela, Mappes, & Vapalahti, 2015; Tersago, Crespin, Verhagen, & Leirs, 2012). The tolerance of rodents to hantavirus infections has mostly been examined by comparing the immune response between reservoir and non reservoir hosts (see for reviews Easterbrook & Klein, 2008, Schountz & Prescott, 2014). Several recent studies have investigated intraspecific variations in immune response of *M. glareolus *to PUUV infections, using samples from geographic regions with high versus low circulation of PUUV in bank voles. In particular, PUUV experimental infections of bank voles originating from NE‐endemic regions (with high circulation and transmission of PUUV) and NE‐free regions (where PUUV is absent or undetectable) have recently been performed (Dubois et al., 2018; Dubois, Castel, et al., 2017). They have shown regional differences in the levels of anti‐PUUV antibodies and of PUUV viral loads in target organs of virus replication (lungs, liver, kidney, and salivary glands). The bank voles from the NE‐endemic area were found to be more tolerant to PUUV infections, as compared to the NE‐free area, suggesting that divergent defense strategies may have locally evolved in these contrasted areas. Additional studies of bank vole populations aimed at characterizing spatial differences in SNP allele frequencies or gene expression levels in candidate genes presumably repressing PUUV replication. These studies reported that tumor necrosis factor (*Tnf*) promoter polymorphisms and variations in *Mx2* gene expression correlated with PUUV distribution in bank vole populations and NE epidemiology (Dubois, Galan, et al., 2017; Guivier et al.., 2010; Guivier, Galan, Henttonen, Cosson, & Charbonnel, 2014).

The recent advent of high‐throughput sequencing technologies now offers the opportunity to go beyond this candidate gene approach and to explore between‐population variations at a genome‐wide scale. To that aim, we combined the sequencing of specific candidate genes and restriction site‐associated DNA (RAD‐seq; see Baird et al., 2008) to characterize genome‐wide patterns of bank vole population differentiation along a spatial transect covering NE‐endemic and NE‐free areas in Sweden. In this country, NE is a reportable disease since 1989 and 10–40 human cases are recorded per 100,000 people each year on average (Olsson, Hjertqvist, Lundkvist, & Hörnfeldt, 2009). Nephropathia epidemica is endemic in the north of the country (Niklasson & LeDuc, 1987; Oscarsson et al., 2016) with about 90% of all human cases being found in the four northernmost counties (Norrbotten, Västernorrland, Västerbotten, and Jämtland). The scarcity of clinical reports and the very weak levels of seroprevalence detected in bank voles in southern Sweden suggest a very low risk of PUUV circulation and transmission below latitude 60 degrees (Dalälven River), although recent studies indicate a potential ongoing range expansion of PUUV around latitude 59° (see, e.g., Borg et al., 2017). This latitudinal pattern is not explained by the reservoir distribution because the bank vole is also common in southern Sweden (Hörling et al., 1996). We therefore hypothesized that bank vole populations from the north of Sweden exhibit tolerance strategies to PUUV. This may favor the circulation and maintenance of PUUV in northern bank vole populations that should, in turn, lead to high spatial risk of NE in this region. Contrastingly, we hypothesized that the low presence and circulation of PUUV in southern bank vole populations might prevent the maintenance or the evolution of tolerance strategies, which should limit the probability for PUUV to establish and persist in this area, making transmission to humans highly unlikely. We therefore characterized genome‐wide patterns of bank vole population differentiation along a north/south transect in Sweden and looked for genomic footprints of divergent selection between NE‐endemic areas in the north and NE‐free areas in the south. To that end, we combined different model‐based methods of genome scan that allowed us to consider several underlying demographic scenarios, as well as putative associations with environmental variables. Last, we tested whether the putative genomic regions responding to divergent selection between the north and south of Sweden were enriched in immune‐related genes. Overall, our study provides preliminary insights into the biological processes that may be involved in *M. glareolus*/PUUV interactions. However, we acknowledge that some methodological and biological issues may limit the interpretation of our results, which must therefore be taken with cautiousness. Accordingly, we strongly recommend that in the future, the immune genes and pathways that we found to be involved in tolerance to PUUV infection should be more thoroughly investigated, in order to confirm their impact on *M. glareolus*/PUUV interactions and to characterize the underlying immune mechanisms at play.

## 2 MATERIALS AND METHODS

### 2.1 Sampling

**Figure 1:**
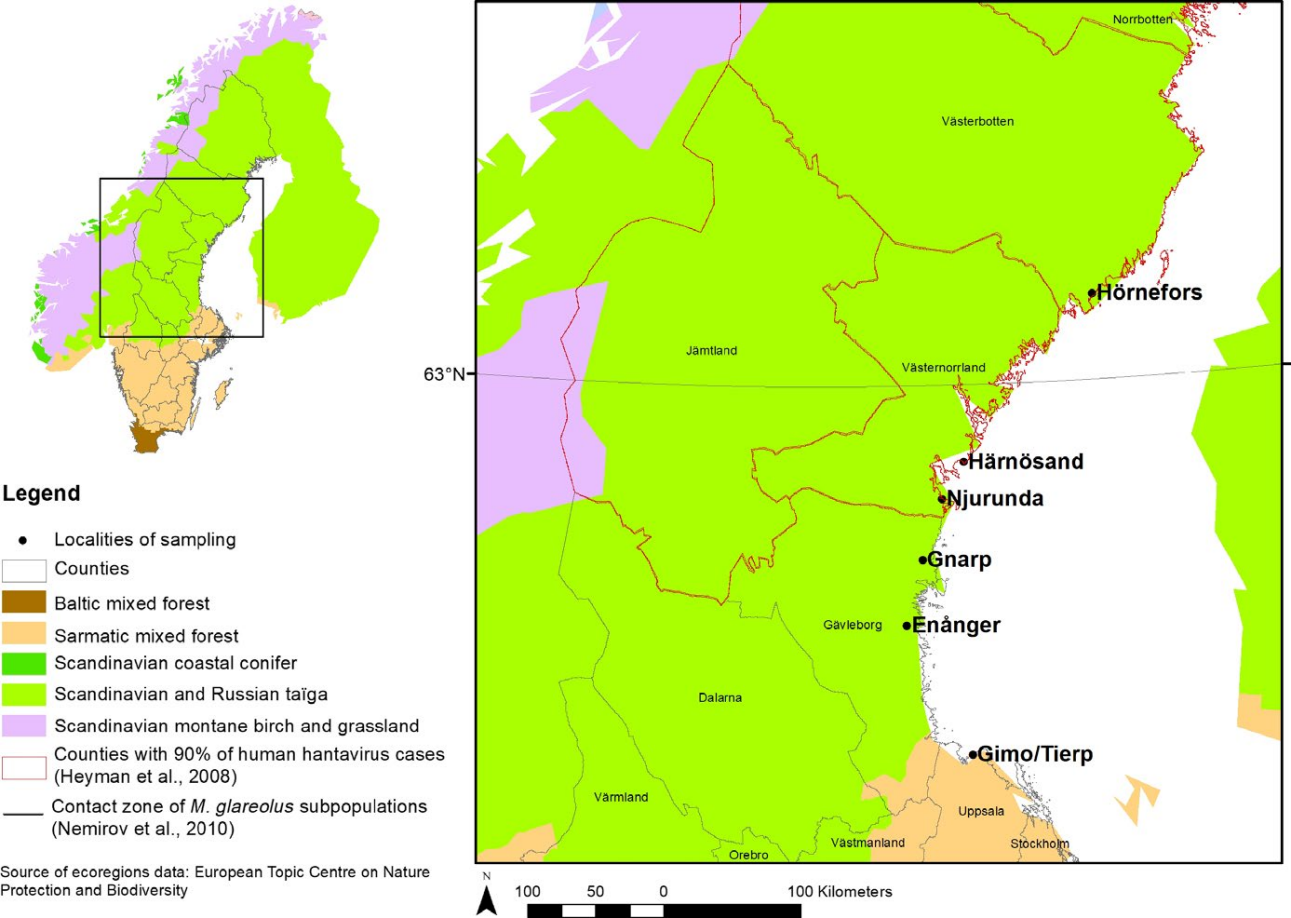
Maps showing the localities of bank vole sampling in Sweden. The red lines delimit the counties with 90% of human hantavirus cases reported. Geographic variations in ecoregions are represented with yellow, orange, and green colors. The contact zone between the two mitochondrial lineages of *Myodes glareolus* is indicated with a black line

Sampling was performed in April and October 2012. Using snap trapping, a total of 257 bank voles were caught in six localities distributed along a transect running from the north of Sweden, which is known to be highly endemic for PUUV, to the south of Sweden, where PUUV is very rare in both bank voles and humans (Figure 1, Table 1; see Olsson, Dalerum, & Hörnfeldt, 2003, Oscarsson et al., 2016, Borg et al., 2017, Gherasim et al., 2015). Because of fieldwork constraints, collected voles were kept on ice and transferred to −20°C freezers, before being processed in the laboratory. Because RNA deteriorates quickly under such storage conditions, PUUV RNA could neither be quantified nor be detected in frozen organs. A piece of hind foot was placed in 95% ethanol for genomic analyses.

**Table 1:** Sampling information

Localities	County	Latitude	Longitude	*N*	Date of sampling	PUUV—human cases Min/Max/Total/Pop. size
Hörnefors	Västerbotten	N63°33′ 50″	E19°47′20″	49	Apr 2012	13/808/2,152/259,239
Härnösand	Västernorrland	N62°29′30″	E17°49′00″	57	Apr 2012	14/391/1,289/242,347
Njurunda	Västernorrland	N62°15′15″	E17°29′45″	20 14	Apr 2012 Oct 2012	14/391/1,289/242,347
Gnarp	Gävleborg	61°51′15″	17°12′10″	47	Oct 2012	4/58/219/276,323
Enånger	Gävleborg	61°25′25″	16°57′58″	28	Oct 2012	4/58/219/276,323
Gimo	Uppsala	60°33′36″	17°50′06″	25 17	Apr 2012 Oct 2012	1/15/95/200,032

Note

Localities of sampling and their administrative county, geographic coordinates (center point from where the voles are trapped, voles are all caught within 1 kilometer from that point), the number *N* of voles trapped, the date of sampling, and the minimum (over 2001–2011), maximum (over 2001–2011) and total number of human cases reported per county between 2001 and 2011 (SMI data) are reported. Note that southern human cases most often correspond to residents spending their holidays in the north of Sweden (Olsson et al., 2009).

We could not use PUUV seroprevalence to assess PUUV‐mediated pressure in the bank vole populations. Indeed, these populations undergo 3‐ to 4‐year population dynamic cycles (Hörnfeldt, 2004) that lead to strong variations of PUUV distribution at fine spatial and temporal scales (Khalil et al., 2014). Dense spatiotemporal surveys are therefore required to gain relevant information on PUUV circulation in these bank vole populations (e.g., Khalil et al., 2017, Ecke, Angeler, Magnusson, Khalil, & Hörnfeldt, 2017). We therefore used NE incidence as a proxy for PUUV‐mediated pressure. In Fennoscandia, human disease risk is highly related to the density of bank vole populations (Khalil et al., 2017; Olsson et al., 2009), and PUUV transmission seems to be density‐dependent in these populations (Voutilainen, Kallio, Niemimaa, Vapalahti, & Henttonen, 2016). Previous studies have also reported high NE incidence in Hörnefors and Härnösand, the two northernmost localities sampled in this study (Ahlm et al., 1998; Olsson et al., 2003; Oscarsson et al., 2016), and the rare southern human cases detected most often corresponded to residents spending their holidays in the north of Sweden (Olsson et al., 2009).

### 2.2 Molecular markers

Genomic DNA was extracted using the EZ‐10 96‐well plate genomic DNA isolation kit for animal samples (Bio Basic Inc.) following the manufacturer recommendations.

#### 2.2.1 Immune‐related candidate genes

We studied the polymorphisms of immune‐related genes that have been shown to be associated with PUUV infections (Tnf promoter, *Mx2*, *Tlr4,* and *Tlr7* genes; see for a review Charbonnel et al., 2014). We evaluated variability at eight single nucleotide polymorphisms that have been previously identified in *M. glareolus *from Fennoscandia (see for details Supporting Information Table S1; Guivier et al., 2010; Dubois, Galan, et al., 2017). Note that we focused on a single SNP within *Tnf* promoter as it had previously been shown to be involved in PUUV/*M. glareolus* interaction (Guivier et al., 2014, 2010). We used KASP genotyping services from LGC company (Genotyping by Allele‐Specific Amplification; Cuppen, 2007) to genotype all sampled voles at these SNPs.

#### 2.2.2 RAD‐tag sequencing

We chose to develop high‐throughput sequencing pools of individuals to reduce sequencing efforts and costs (Gautier et al., 2013; Schlötterer, Tobler, Kofler, & Nolte, 2014). This approach provided genomic data at the population level, which prevented us from running genome‐wide association analyses at the individual level. Six equimolar pools (from 35 to 37 individuals per locality) were realized after DNA quality control using NanoDrop, 1.5% agarose gel electrophoresis, and Qubit® 2.0 Fluorometer (Invitrogen) quantification.

RAD sequencing was performed following the protocol designed by Etter et al. (2011) and modified by Cruaud et al. (2014). Briefly, DNA pools were digested with 8‐cutter restriction enzyme *Sbf*I (21 700 sites predicted following the radcounter_v4.xls spreadsheet available from the UK RAD Sequencing Wiki (www.wiki.ed.ac.uk/display/RADSequencing/Home). For each locality, we built four independent libraries to avoid methodological biases. Digested DNA pools were ligated to a modified Illumina P1 adapter containing locality‐specific, 5‐ to 6‐bp‐long multiplex identifiers (MIDs). All MIDs differed by at least three nucleotides to limit erroneous sample assignment due to sequencing error (Additional file, Supporting Information Table S2). The 24 libraries were then pooled and sheared by sonication using S220 ultrasonicator (Covaris, Inc.). Genomic libraries were size‐selected for 300–500 bp by agarose gel excision. P2 adapter were then ligated and fragments containing both adapters (P1 and P2) were PCR‐enriched during 15 cycles. Libraries were sequenced on an Illumina HiSeq 2000 platform (v3 chemistry) using 2 × 100 bp paired‐end sequencing. Illumina sequencing was performed at the GenePool Genomics Facility (University of Edinburgh, UK).

Sequence reads from Illumina runs were demultiplexed and quality‐filtered using the *process_radtags* program from the Stacks package version 0.99994. Ambiguous MIDs and low‐quality reads (Phred < 33) were discarded from further analyses. Sequences were trimmed to 85 nucleotides (position 5–90 after the MIDs for the reads 1; position 1–85 for the reads 2).

Because no reference genome assembly was available for *M. glareolus*, we needed to build a de novo RAD assembly. To that end we first assembled reads 1 per sample with the *ustacks* program from the Stacks package and default options, except for (a) the minimum depth of coverage required to create a stack (‐m option) that was set to 2; (b) the maximum distance in nucleotides allowed between stacks (‐M option) that was set to 3; and (c) the maximum distance allowed to align secondary reads to primary stacks (‐N option) that was set to 2. The resulting set of loci were then merged into a catalog of loci by the *cstacks* program from the Stacks package run with default options except for the number of mismatches allowed between sample tags to form stacks (‐n option) that was set to 2. For each of the obtained read 1 contigs (i.e., RAD loci), we further assembled the associated reads 2 using *CAP3* (Hang & Madan, 1999) ran with default options except for (a) the segment pair score cutoff (‐i option) that was set to 25; (b) the overlap length cutoff (‐o option) that was set to 25; and (c) the overlap similarity score cutoff (‐s option) that was set to 400. If a single contig was produced after a first *CAP3* run, this was retained only if it was associated with <5% remaining singleton sequences and supported by more than 40 reads. If several contigs were produced, CAP3 was run a second time (using the same options as above) to try to assemble all contigs into a single one, which was in this case retained for further analyses. If read 2 contig overlapped with their corresponding read 1 contig, as assessed with the *blastn* program from the BLAST+ v2.2.26 suite (*e*‐value < 1e‐10 and percentage of identity above 95), both contigs were concatenated. Otherwise, fifteen “Ns” were inserted between both contigs.

Sequence reads were aligned to this assembly using the programs *aln* and *sampe *implemented in *bwa* 0.5.9 and ran with default options. The resulting *bam* files were then jointly analyzed with the *mpileup* program from the Samtools v0.1.19 suite. We used default options except for the minimum mapping quality for alignment (‐q option) that was set to 20. The mpileup file was further processed using a custom awk script to perform SNP calling and derive read counts for each alternative base (after discarding bases with a Base Alignment Quality score <25) as previously described (Gautier, 2015; Gautier et al., 2013). A position was considered variable if (a) it had a coverage of >5 and <500 reads in each pool; (b) only two different bases were observed across all six pools; and (c) the minor allele was represented by at least one read in two different pool samples. Note that triallelic positions for which the two most frequent alleles satisfied the above criteria with the third allele represented by only one read were included in the analysis as biallelic SNPs (after filtering the third allele as a sequencing error).

### 2.3 Characterization of genetic diversity and population structure

We performed genetic diversity analyses on the genotypes inferred at candidate gene SNPs. Gene diversities within (*H*_o_) and among (*H*_e_) individuals, as well as the inbreeding coefficient *F*_IS_, were estimated using Genepop v4.2 (Rousset, 2008). Deviation from Hardy–Weinberg equilibrium was assessed using exact tests implemented in Genepop v4.2. Linkage disequilibrium (LD) was estimated using the program Linkdos implemented in Genetix v4.05 (Belkhir, Borsa, Chikhi, Raufaste, & Bonhomme, 1996–2004). Significance was assessed using permutation tests.

For RAD‐seq markers, we characterized the extent of genetic differentiation between populations using *F*_ST_. Yet, because computing *F*_ST_ from read counts as if they were allele counts ignores the extra sampling error brought by the random sampling of reads from the gene pool during sequencing, we used the method‐of‐moments estimator developed by Hivert, Leblois, Petit, Gautier, and Vitalis (2018) for Pool‐seq data, based on an analysis‐of‐variance framework. Next, we estimated the scaled covariance matrix of population allele frequencies using the algorithm implemented in BayPass (Gautier, 2015). A principal component analysis (PCA) was performed on this matrix using the FactoMineR library in R to visualize the patterns of population structure. All SNPs were included, as the estimation of the covariance matrix in BayPass is expected to be robust to the presence of markers evolving under selection (see Gautier, 2015). Finally, we tested for an isolation‐by‐distance pattern following Rousset (1997) by analyzing the relationship between pairwise genetic distances, estimated as *F*_ST_/(1 − *F*_ST_), and the logarithm of geographic distances using a Mantel test implemented in R (R Development & Team, 2018).

### 2.4 Detecting putative footprints of selection based on genetic differentiation

Genome scans for adaptive differentiation were performed using two complementary methods: SelEstim version 1.1.3 (Vitalis, Gautier, Dawson, & Beaumont, 2014) and BayPass version 2.1 (Gautier, 2015), which both handle Pool‐seq data. Although these methods both aim at characterizing markers showing outstanding differentiation, relatively to the rest of the genome, they rely on different premises. The analyses were performed on all six populations altogether and included all candidate loci polymorphisms and RAD‐seq markers. SelEstim (Vitalis et al., 2014) is based on a diffusion approximation for the distribution of allele frequencies in an island model with selection. In particular, SelEstim assumes that each and every locus is targeted by selection to some extent, and estimates the strength of selection at each locus and in each population. SelEstim uses a Markov chain Monte Carlo (MCMC) algorithm to sample from the posterior distribution of the model parameters, in a hierarchical Bayesian framework. To prevent any convergence issue with SelEstim, the final dataset was transformed by randomizing the reference allele for each and every locus, using the randomize.reference.allele() R function provided in the package. For each analysis, twenty‐five short pilot runs (1,000 iterations each) were set to adjust the proposal distributions for each model parameter and, after a 100,000 burn‐in period, 100,000 updating steps were performed. Samples were collected for all the model parameters every 40 steps (thinning interval), yielding 2,500 observations. Three different SelEstim analyses were run on the same data, to assess convergence using the Gelman–Rubin diagnostic implemented in the CODA package for R (Plummer, Best, Cowles, & Vines, 2006). Provided convergence was assessed, one replicate analysis was picked at random for the rest of the study. Candidate markers under selection were selected on the basis of the distance between the posterior distribution of the locus‐specific coefficients of selection and a “centering distribution” derived from the distribution of the genome‐wide parameter that accounts for the variation of selection strength across loci. SelEstim uses the Kullback–Leibler divergence (KLD) as a distance between these two distributions, which is calibrated using simulations from a posterior predictive distribution based on the observed data (Vitalis et al., 2014). Here, we generated a pseudo‐observed dataset (POD) from the inference model, with hyperparameters set to their respective posterior means, using a rejection‐sampling algorithm. A SelEstim analysis of that POD provided an empirical distribution of KLD values, from which quantiles were computed to calibrate the KLD observed for each locus in the original data. Hereafter, we report candidate markers with KLD values above the 99.9% quantile of the so‐obtained empirical distribution of KLD (although the results based on the 99.95% and 99.99% quantiles are also provided).

There are two locus‐ and population‐specific parameters in SelEstim: the coefficient of selection σ*_ij_* and a Boolean indicator variable (κ_ij_) that indicates which allele is selected for in the *i*th deme, at the *j*th locus. If the reference (respectively the alternative) allele is selected for in one deme, then the posterior mean of κ_ij_ is shifted toward 0 (resp. 1) (see Vitalis et al., 2014). Therefore, the posterior distribution of κ_ij_ across populations carries information on selection acting differentially between populations (see Nouhaud et al., 2018). In order to appreciate the differential strength of selection at any locus across populations, we therefore used the composite parameter ζ*_ij_* ≡ 2 σ*_ij_* (κ*_ij_* − 0.5), computed from the posterior means of σ*_ij_* and κ_ij_. This composite parameter tends to 0 when κ*_ij_* tends to 0.5 (which is expected for a neutral marker); it tends to −σ*_ij_* when κ*_ij_* tends to 0 (which is expected when the reference allele is selected for); it tends to σ*_ij_* when κ*_ij_* tends to 1 (which is expected when the alternative allele is selected). Broadly positive or negative values of ζ_ij_ are therefore expected when selection is strong at one locus in one population. Then, we examined the correlation of ζ*_ij_* with some environmental variables.

Next, we used the core model implemented in the software package BayPass (Gautier, 2015) to characterize the genetics structure of populations and identify outlier loci. This hierarchical Bayesian model, which extends and outperforms the approach by Coop, Witonsky, Rienzo, and Pritchard (2010) and Gunther and Coop (2013), relies on the estimation of the (scaled) covariance matrix of population allele frequencies, which is known to be informative about demographic history. Therefore, contrary to SelEstim, BayPass is not limited by the oversimplification of the underlying demographic model. To identify SNPs targeted by selection, we used BayPass to estimate the statistic *X* ^T^ *X*, which might be interpreted as a locus‐specific analogue of *F*_ST_, explicitly corrected for the scaled covariance of population allele frequencies. To define a significance threshold for the *X* ^T^ *X* statistic, we estimated the posterior predictive distribution of this statistic under the core model, by generating and analyzing a POD made of 20,000 SNPs (Gautier, 2015). We checked that the scaled covariance matrix of population allele frequencies estimated from the POD was close to the matrix estimated from our data (*FMD* distance = 0.088; see Förstner and Moonen (2003) for a definition of this metric). The decision criterion for identifying *X* ^T^ *X* outliers was defined from the quantiles of the *X* ^T^ *X* distribution of the POD analysis.

### 2.5 Detecting putative footprints of selection based on environmental variables

We used BayPass to test for associations between allele frequencies and environmental variables presumably related to PUUV epidemiology, while controlling for demography. The standard covariate model (STD) in BayPass assumes a linear effect of the environmental variable on allele frequencies. Association of SNPs with environmental variables was assessed using empirical Bayesian *p*‐values (eBP). Roughly speaking, for a given SNP, the empirical Bayesian *p*‐value measures to which extent the posterior distribution of the regression coefficient excludes 0 (Gautier, 2015). In order to compute the eBP, we used the importance sampling algorithm implemented in BayPass (eBP_is_) to estimate the moments of the posterior distribution of the regression coefficients. We calibrated eBP_is_ by generating and analyzing a POD.

Environmental variables related to PUUV prevalence in human (number of NE cases), climate, forest composition and shape, and soil water content, were selected with regard to PUUV ecology in Europe as they should reflect PUUV distribution in Sweden (Zeimes et al., 2015; Zeimes, Olsson, Ahlm, & Vanwambeke, 2012) (Table 2). Except for the number of NE cases that was only available per county, variables were computed within an area covering a circular radius of 3 km around each sampling site (ArcGIS 10.1), which is an acceptable estimate of vole dispersal capacity (Le Galliard, Rémy, Ims, & Lambin, 2012). To summarize climate variation, we used the minimum temperature in winter (December, January, and February), the maximum temperature in summer (June, July, and August), the percentage of the area covered by snow, and the annual precipitation. Land use was characterized by forest types (the proportion in the 3‐km buffer of forest, coniferous, broadleaved, and mixed forest) and by tree species (the volume of spruce and pine and their standard deviation). Forest patches metrics (computed with FRAGSTATS, version 4; https://www.umass.edu/landeco/research/fragstats/fragstats.html) were averaged in the 3‐km buffer and included the contiguity index, the shape index (a shape index of one represents the most compact shape, upper than one, a more complex shape), and the perimeter. Finally, the soil water index (SWI) representing the soil moisture conditions was also included.

**Table 2:** Environmental parameters and their potential impacts on *Myodes glareolus *and Puumala

Variables	Bank voles	Virus	Resolution	Units	Sources
PUUV presence					
Total number of human cases between 2001 and 2011 (SWI dataset)		x	County		
Climatic variables					
Minimum temperature in winter (MinTempWinter)	x	x	0.0083° 1950–2000	°C	WorldClim
Maximum temperature in summer (MaxTempSummer)		x	0.0083° 1950–2000	°C	WorldClim
Snow cover (Snow)	x	x	0.005° 2000–2008	Area percentage	MODIS
Annul precipitation (Pp)	x	x	0.0083° 1950–2000	mm	WorldClim
Forest and soil indices					
Proportion of forest	x		100 m	Area percentage	Corine 2006 (EEA)
Proportion of broadleaved forest	x		100 m	Area percentage	Corine 2006 (EEA)
Proportion of coniferous forest	x		100 m	Area percentage	Corine 2006 (EEA)
Proportion of mixed forest	x		100 m	Area percentage	Corine 2006 (EEA)
Mean volume of spruce	x		25 m	m^3^/ha	SLU Skogskarta
Standard deviation of the volume of spruce	x		25 m	m^3^/ha	SLU Skogskarta
Mean volume of pine	x		25 m	m^3^/ha	SLU Skogskarta
Standard deviation of the volume of pine	x		25 m	m^3^/ha	SLU Skogskarta
Average contiguity index of forest patches	x		1:20,000	Relative unit	Lantmäteriet
Average shape index of forest patches	x		1:20,000	Relative unit	Lantmäteriet
Perimeter of the forest patches	x		1:20,000	m	Lantmäteriet
Soil water index (SWI)	x	x	25 km 2007–2010	Relative unit	TU‐WIEN

Note

“x” indicates that the environmental factor may affect bank voles’ abundance or virus survival outside the host.

**Figure 2:**
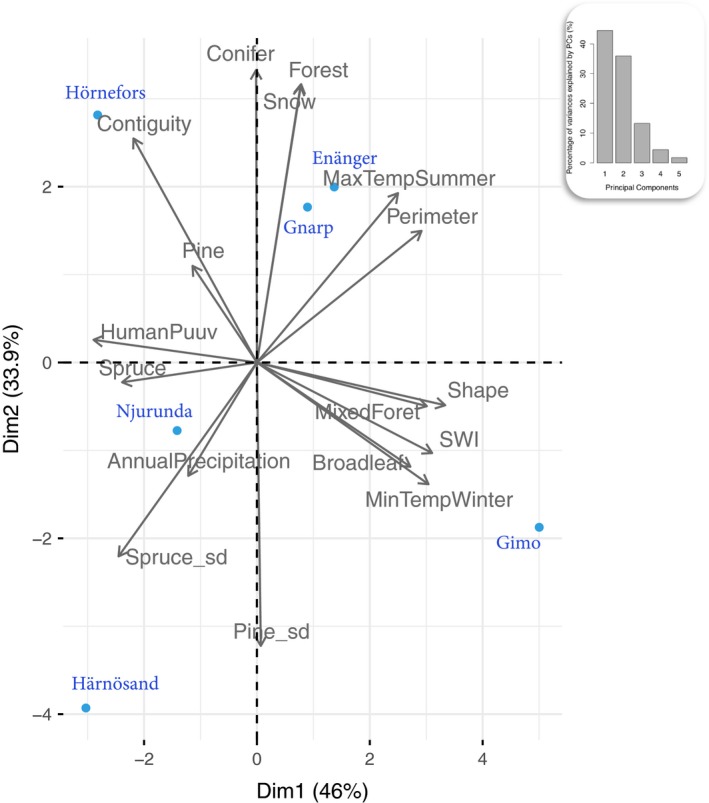
Principal component analysis (PCA) plot of the environmental data (black labels) and of the six populations (blue labels) analyzed. “Dim 1” and “Dim 2” indicate the proportions of variance explained by each axis. Details about environmental variables are provided in Table 2. The inset graph indicates the proportion of variance explained by each subsequent eigenvalues of the covariance matrix of the data

To reduce the dimensionality of these environmental data, we assessed SNP–environment associations using the two first principal components (PC) from a PCA that included all 15 variables (Figure 2). These new synthetic variables explained respectively 46.0% and 33.9% of the total variance. PC1 represented an environmental, latitudinal gradient. Along this axis, sampling localities were ranked from northern localities (negative values) exhibiting high numbers of human PUUV infection and large volume of spruce forests, to southern localities (positive values) with mixed or broadleaved forests, low mean winter and high maximum summer temperatures. PC2 strongly opposed the two northernmost sampled localities, with Hörnefors (positive values) being characterized by large volume of contiguous coniferous forest and high snow coverage, and Härnösand (negative values) being more fragmented.

### 2.6 Annotations and gene ontology analysis

#### 2.6.1 Annotation of contig sequences

In the absence of a published high‐quality genome assembly and gene annotation for *M. glareolus*, we compared the RAD contigs to the genome of *Mus musculus* (build GRCm38/mm10). We downloaded (date: 19/01/2018) the GFF3 gene annotation and the sequences of the 22 mouse chromosomes, 111,462 cDNAs and 64,553 proteins from ENSEMBL (release 91; https://www.ensembl.org/) and blasted the RAD contigs against these sequences. We used blastn for genome and cDNA (version 2.5.0+, with threshold e‐value of 1e‐5, minimum alignment percent identity of 70%, and options ‐show_gis, ‐outfmt 6, and –num_alignments 1), and blastx for proteins (with the same parameters as blastn but without identity criterion). Regarding the whole‐genome alignment, all RAD outliers being aligned on a mouse gene were identified by considering the gene coordinates being present in the GFF3 gene structure file.

#### 2.6.2 Enrichment analysis of outlier candidates

For enrichment analyses, the Blast2GO program v5.0.13 (Conesa et al., 2005) was run to search the Gene Ontology (GO) database (version 02/24/2018, Ashburner et al., 2000) for overrepresented GO terms. For this purpose, the ENSEMBL gene id for all RAD outliers that were aligned on a mouse gene was extracted along with the gene id of the candidate *Tlr7 *(ENSMUSG00000044583). As background set the BioMart annotations for 53,946 mouse genes were obtained online via the Blast2GO interface. The overrepresentation of outlier GO terms was tested using Fisher's exact test and a *p*‐value threshold of 0.05 corrected using FDR (Benjamini & Hochberg, 1995). Furthermore, we ran the web program REVIGO (web version of 02/14/2018, Supek, Bošnjak, Škunca, & Šmuc, 2011) to group significantly enriched Gene Ontology (GO) terms into GO categories and to summarize the GO terms to common terms. We applied the SimRel semantic similarity measure with the default GO term similarity of 0.7, associated corrected *p*‐values to the enriched GO terms, and the *M. musculus* database (Supek et al., 2011). In addition, the Reactome, KEGG, and INOH databases were searched using InnateDB analysis webtool (Breuer et al., 2013) for overrepresented metabolic pathways which were associated to the mouse gene ids of the aligned outliers. A metabolic pathway was considered as significantly overrepresented if the associated *p*‐value was <0.05 after FDR correction.

The above analyses were also conducted using genome assemblies of two alternative species: the prairie vole, *Microtus ochrogaster* (ENSEMBL assembly MicOch1.0), and the Chinese hamster, *Cricetulus griseus* (ENSEMBL assembly CriGri_1.0). Given the low quality of annotation for these two genomes, we could not identify any gene enrichment, and we therefore present the results based on the *M. musculus* genome assembly only.

## 3 RESULTS

### 3.1 RAD‐tag sequencing

Sequencing of the 24 RAD libraries (6 localities and 4 replicates) generated 340,692,418 reads, with an average of ca. 13.5 million reads per multiplex identifiers (MIDs). The number of sequences generated per locality and MID ranged between ca. 10.1 and ca. 15.1 million reads. After trimming sequences to 85 bp and after filtering for quality, an average of ca. 12.6 million reads per MID (representing 93.5% of the total) were retained. Read 1 assembling produced 151,522 contigs. The first *CAP3* assembling run of the associated reads 2 produced 46,474 unique contigs represented by more than 40 sequences, and among which 46,471 had <5% singleton. The second *CAP3* assembling run enabled to provide 69,777 read 2 contigs. We found a single significant alignment between read 1 and read 2 contigs in 59,242 cases and no significant overlap in 10,495 cases. The other 38 cases corresponded to a complete alignment of read one contig with read two contig (one case), an alignment of read one contig within read two contig (two cases) and multiple significant alignment (35 cases). The resulting assembly finally consisted of 69,777 contigs spanning 38,482 Mb (average contig size equal to 551.5 bp, [209–891]). Reads were aligned to this reference contig dataset and 485,182 SNPs were detected (QC > 25, depth range: 5–500×). Among them, 95,988 SNPs distributed on 70,699 contigs were kept according to the more stringent criteria described before.

### 3.2 Descriptive statistics

#### 3.2.1 Candidate gene diversity

The eight SNPs within Tnf promoter and *Tlr4*, *Tlr7*, and *Mx2* genes were successfully genotyped in 250 Swedish bank voles using the KASP genotyping. Not surprisingly, significant linkage disequilibrium (LD) was observed between most SNPs located within genes (*p* < 0.05): *Tlr4*‐exon3 776 and *Tlr4*‐exon3 1146, *Tlr4*‐exon3 1662, *Tlr4*‐exon3 1687; *Tlr4*‐exon3 1146 and *Tlr4*‐exon3 1662, *Tlr4*‐exon3 1687; *Tlr4*‐exon3 1662 and *Tlr4*‐exon3 1687. We did not detect any linkage disequilibrium between SNPs located in different genes.

Estimates of diversity indices per SNP and per sampling locality are given in Table 3. Deviation from Hardy–Weinberg equilibrium was observed in most localities for *Tnf* promoter (SNP *Tnf* ‐296; see Guivier et al., 2010) with significant deficits in heterozygotes detected in Hörnefors, Härnösand, Njurunda, and Gimo (*p* < 0.05). Moreover, significant departures from Hardy–Weinberg expectations were observed in Gimo for two SNPs after correcting for multiple tests.

**Table 3:** Diversity indices per sampling locality and SNP

SNP	Indices	Localities					
		Hörnefors	Härnösand	Njurunda	Gnarp	Enånger	Gimo
*Mx2*_14_162	*H*_e_	0.4639	0.1759	0.000	0.000	0.000	0.000
	*H*_o_	0.4694	0.1579	0.000	0.000	0.000	0.000
	*F*_IS_	−0.012	0.103	–	–	–	–
*Tlr4*_667	*H*_e_	0.000	0.000	0.0294	0.1311	0.1726	0.5065
	*H*_o_	0.000	0.000	0.0294	0.1389	0.1875	0.2703
	*F*_IS_	–	–	0.000	−0.061	−0.088	**0.470 (0.007)**
*Tlr4*_776	*H*_e_	0.0600	0.0517	0.2950	0.4261	0.4583	0.2755
	*H*_o_	0.0612	0.0526	0.2941	0.4286	0.5000	0.1622
	*F*_IS_	−0.021	−0.018	0.003	−0.006	−0.093	**0.415 (0.034)**
*Tlr4_*1146	*H*_e_	0.0791	0.0517	0.2950	0.4409	0.4583	0.2755
	*H*_o_	0.0816	0.0526	0.2941	0.4167	0.5000	0.1622
	*F*_IS_	−0.032	−0.018	0.003	0.056	−0.093	**0.415 (0.033)**
*Tlr4*_1662	*H*_e_	0.0600	0.0517	0.3021	0.4343	0.4583	0.2936
	*H*_o_	0.0612	0.0526	0.3030	0.4054	0.5000	0.1892
	*F*_IS_	−0.021	−0.018	−0.003	0.067	−0.093	0.359
*Tlr4*_1687	*H*_e_	0.0412	0.0517	0.2950	0.4409	0.4583	0.2755
	*H*_o_	0.0417	0.0526	0.2941	0.4167	0.5000	0.1622
	*F*_IS_	−0.011	−0.018	0.003	0.056	−0.093	**0.415 (0.031)**
*Tlr7*_2593	*H*_e_	0.0000	0.3552	0.1383	0.4261	0.5079	0.0000
	*H*_o_	0.0000	0.1404	0.1471	0.3143	0.3750	0.0000
	*F*_IS_	–	**0.607**	−0.065	0.265	0.265	–
*Tnfp*_296	*H*_e_	0.4033	0.4988	0.3652	0.2950	0.2285	0.4832
	*H*_o_	0.0204	0.0175	0.1765	0.2941	0.1935	0.2432
	*F*_IS_	**0.950 (<10^−^^5^)**	**0.965 (<10^−^^5^)**	**0.521 0.006**	0.003	0.155	**0.500 (0.004)**

Note

*H*_e_ is the gene diversity among individuals within samples; *H*_o_ is the gene diversity within individuals; *F*_IS _is the inbreeding coefficient. All these parameters were estimated with Genepop. Values in bold indicate significant deviation from Hardy–Weinberg equilibrium. *p*‐values are indicated in brackets.

#### 3.2.2 Characterization of population structure

**Figure 3:**
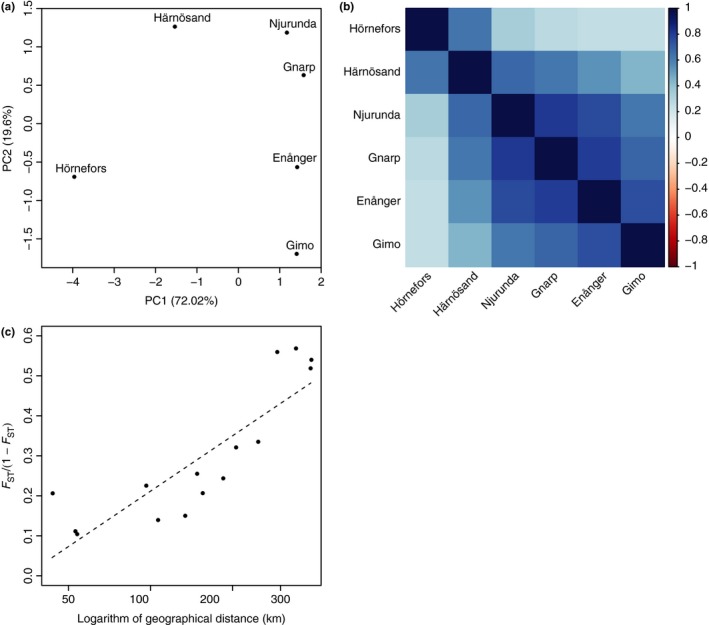
Graphical representations of the *Myodes glareolus* population genetic structure based on the 95,988 SNPS. (a) Principal component analysis (PCA) based on the variance covariance matrix of the six bank vole populations studied, estimated using BayPass and based on the 95,988 SNPs included in the statistical analyses. (b) Representation of the scaled covariance matrix as estimated from BayPass under the core model with  = 1. (c) Multilocus estimates of pairwise genetic differentiation, computed as *F*_ST_/(1 − *F*_ST_), plotted against logarithm of geographic distances between all possible pairs of bank vole populations. The regression is represented as a dashed line

The multilocus *F*_ST_ between pairs of populations ranged from 0.091 to 0.361, and the overall differentiation among populations was estimated as *F*_ST_ = 0.212 (see details in Supporting Information Table S3). We found a significantly positive correlation between pairwise genetic distances, estimated as *F*_ST_/(1 − *F*_ST_), and the logarithm of geographic distance (Mantel test; *p* = 0.0014). The PCA performed on the covariance matrix of population allele frequencies revealed a strong differentiation between the two northernmost populations, Hörnefors and Härnösand, and all other populations on the first axis. The second axis differentiated the southern population of Gimo from all other populations (Figure 3a). The scaled covariance matrix of population allele frequencies estimated with BayPass was also consistent with a strong differentiation between Hörnefors and Härnösand populations on the one hand, and more southern populations on the other hand (Figure 3b), as well as with an isolation‐by‐distance pattern (Figure 3c).

### 3.3 Signatures of selection based on genetic differentiation

#### 3.3.1 SelEstim

**Figure 4:**
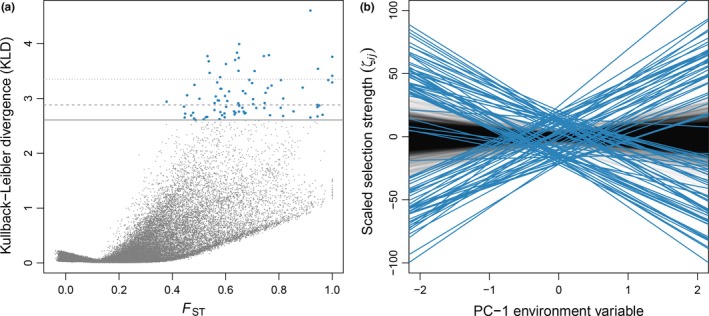
SelEstim analysis of 95,988 SNPs in the six bank vole populations. (a) Kullback–Leibler divergence (KLD) as a function of *F*_ST_ estimates for all SNPs. Horizontal lines indicate the 99.90% (solid), 99.95% (dashed), and 99.99% (dotted) quantiles computed using the KLD calibration procedure. Outlier SNPs detected at the 99.90% threshold value are depicted in blue. (b) Regression of the locus‐ and population‐specific coefficient of selection, scaled as: ζ_ij_ ≡ 2 σ_ij_ (κ_ij_ − 0.5) (see the Materials and Methods section), to the coordinates of the first principal components of the environmental variables. The blue lines correspond to outlier SNPs detected at the 99.90% threshold value, and the thin gray lines correspond to all other markers

The Gelman–Rubin diagnostic was equal to 1.06 for the highest‐level parameter in the SelEstim model, which indicates that the three independent chains converged satisfactorily to the target distribution. One replicate analysis was therefore picked at random for the rest of the study. The 99.9% quantile of the posterior predictive distribution of the KLD (based on pseudo‐observed data) equaled 2.61. We found a total of 86 SNPs, representing 78 unique contigs and the candidate gene *Tlr7*, with a KLD estimate equal to or larger than this threshold (48 SNPs were identified as outliers using the 99.95% quantile threshold, and 17 at the 99.99% threshold). All these outliers showed high *F*_ST_ estimates compared with the background *F*_ST_ (Figure 4a). We found that for most outliers, the composite parameters of selection ζ_ij_ correlated with the first principal component of the environmental variables (Figure 4b).

#### 3.3.2 BayPass

**Figure 5:**
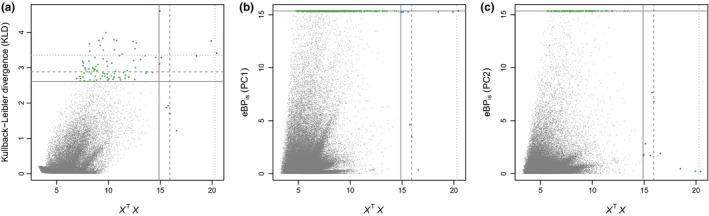
Outlier detection based on 95,988 SNPs in the six bank vole populations using BayPass. (a) Correlation between the Kullback–Leibler divergence (KLD) for all SNPs and the *X* ^T^ *X* statistics estimated using BayPass. Horizontal lines correspond to the 99.90% (solid), 99.95% (dashed), and 99.99% (dotted) quantiles calculated from the KLD calibration procedure. Vertical lines correspond to the 99.90% (solid), 99.95% (dashed), and 99.99% (dotted) quantiles of the *X* ^T^ *X* distribution calculated using simulations from a predictive distribution based on the observed dataset. SNPs that were detected at the 99.90% threshold value of KLD with SelEstim are depicted in green. SNPs that were detected at the 99.90% threshold value of *X* ^T^ *X* with BayPass are depicted in blue. SNPs that were detected by both SelEstim and BayPass are depicted in red. (b) Correlation between the statistics eBP (with the environmental synthetic variable being PCA axis 1) and *X* ^T^ *X* estimated using BayPass. Horizontal and vertical lines correspond to the 99.90%, 99.95% and 99.99% quantiles calculated from the simulations described above. (c) Correlation between the statistics eBP (with the environmental synthetic variable being PCA axis 2) and *X* ^T^ *X* estimated using BayPass. Horizontal and vertical lines correspond to the 99.90%, 99.95% and 99.99% quantiles calculated from the simulations described above

Considering the core model implemented in BayPass (i.e., the covariable‐free approach), we found 10 outlier SNPs, belonging to nine unique contigs (*X* ^T^ *X* > 14.88, 99.9% quantile). Six SNPs corresponding to five unique contigs were common with the SelEstim analysis (Figure 5a). Considering the 99.95% and the 99.99% quantiles we found five and one outlier SNP(s), respectively. Out of these, three (respectively zero) were common with the SelEstim analysis (Additional file, Supporting Information Figure S1).

### 3.4 Signatures of selection based on environmental correlations

Using the STD model in BayPass (which allows the evaluation of associations between SNP allele frequencies and environmental variables), we found 483 SNPs (belonging to 413 unique contigs) at the 99.90% threshold, with strong association signals (eBP_is_ > 15.35). A total of 395 SNPs — corresponding to 339 unique contigs — showed significant association with the first principal component of the environmental variables (Figure 5b). Among them, 26 contigs were previously detected using SelEstim only (24 contigs) or SelEstim and BayPass core model (2 contigs). Ninety SNPs—corresponding to 77 unique contigs—were associated with the second principal component of the environmental variables (Figure 5c). Four of these contigs were previously detected as outliers: three from the BayPass STD model with the first PC, and another one from the SelEstim analysis. None of these SNPs were found as outliers in the BayPass core model. Results obtained with other threshold values are provided in Additional file (Supporting Information Figure S1).

### 3.5 Annotation and gene ontology analysis

#### 3.5.1 Annotation of contig sequences

We could annotate 44.4% (*n* = 30,973) of the 69,777 bank vole contigs using the *M. musculus* genome sequence from ENSEMBL. These contigs covered all 22 mouse chromosomes. Moreover, about 21% (*n* = 14,509) of the 69,777 bank vole contigs were located in 5,977 mouse protein‐coding genes (cDNAs). Out of these, 9,794 (14%) bank vole RAD contigs were actually located in the coding sequence of 4,853 mouse proteins (the other RAD contigs were more likely located in UTR termini of the cDNA).

#### 3.5.2 Annotation, function and gene enrichment analysis of outlier candidate genes

Annotation and pathway enrichment analyses were performed on the 468 bank vole outlier contigs detected by at least one of the genome‐scan methods. Among them, 191 were aligned to the mouse genome, covering 20 mouse chromosomes. In total, 108 outliers—including the *Tlr7* candidate gene—matched to 104 mouse protein‐coding genes (Additional file, Supporting Information Table S4). We found GO annotations for 95 of them. Fourteen genes had GO annotations related to immunity (*Il12rb1, Lbp, Ptprc, Tlr7, Tnfsf10, Sema3d, Rbpj, Atm, Pibf1, Fes, Bmpr1b, Fermt3, RpS6ka3, and RpS6kb1*). They were either detected either by the algorithm of SelEstim or by that of the STD model in BayPass (Table 4).

**Table 4:** Outlier SNPs identified using at least one of the three methods implemented to detect signatures of selection: SelEstim, BayPass and BayPass with environmental variables (respectively when associations were found with PC1 or PC2). Only SNPS that belonged to contigs that aligned to the mouse genome and corresponded to genes coding for proteins related to immunity or involved in responses to hantavirus infections are indicated. Gene name and description were obtained in ENSEMBL. Pathways are indicated following KEGG or Reactome results

Gene ID (ENSEMBL)	SNP (MRK_RAD)	Consensus Sequence	Gene abbreviation and synonyms	Gene name and description	Method of outlier detection	Pathway
ENSMUSG00000026395	13,196	C134235	Ptprc, B220, CD45R, Cd45, L‐CA, Ly‐5, Lyt‐4, T200, loc	Protein tyrosine phosphatase, receptor type C; protein tyrosine‐protein phosphatase required for T‐cell activation through the antigen receptor.	KLD	Cell adhesion molecules (CAMs) Fc gamma R‐mediated phagocytosis T‐cell receptor signaling pathway
ENSMUSG00000053158	12,626	C132789	Fes	Feline sarcoma oncogene	KLD	Developmental biology pathway; axon guidance pathway; IL‐3, IL‐4, IL‐6, and IL‐11 pathways
ENSMUSG00000044583		Candidate gene	Tlr7	Toll‐like receptor 7; key component of innate and adaptive immunity	KLD eBP‐1	Toll‐like receptor signaling pathway
ENSMUSG00000000791	89,604	C84915	Il12rb1, CD212, IL‐12R[b]	Interleukin‐12 receptor, beta 1; functions as an interleukin receptor which binds interleukin‐12 with low affinity and is involved in IL‐12 transduction	eBP‐1	Cytokine–cytokine receptor interaction JAK–STAT signaling pathway Interleukin signaling pathway
ENSMUSG00000016024	94,448	C96304	Lbp, Bpifd2, Ly88	Lipopolysaccharide binding protein; binds to the lipid A moiety of bacterial lipopolysaccharides (LPS) and acts as an affinity enhancer for CD14	eBP‐1	NF‐kappa B signaling pathway Toll‐like receptor signaling pathway Salmonella infection Tuberculosis
ENSMUSG00000024965	71,214	C41693	Fermt3, C79673, Kindlin3	Fermitin family homolog 3 (Drosophila); plays a central role in cell adhesion in hematopoietic cells and acts by activating the integrin beta‐1‐3 required for integrin‐mediated platelet adhesion and leukocyte adhesion to endothelial cells	eBP‐1	Platelet activation
ENSMUSG00000039304	42,318	C217696	Tnfsf10	Tumor necrosis factor (ligand) superfamily, member 10	eBP‐1	Cytokine–cytokine receptor interaction, apoptosis, natural killer cell‐mediated cytotoxicity
ENSMUSG00000040254	81,126	C65031	Sema3d	Semaphorin 3D	eBP‐1	Cell adhesion molecules and their ligands, axon guidance pathway, immunoglobulin superfamily
ENSMUSG00000039191	13,804	C13591	Rbpj	Recombination signal binding protein for immunoglobulin kappa J region	eBP‐1	Notch signaling pathway, disease pathway, Th1 and Th2 cell differentiation
ENSMUSG00000034218	86,924	C78794	Atm	Ataxia telangiectasia mutated family protein	eBP‐1	NF‐kappa B signaling pathway
ENSMUSG00000022064	94,686	C96906	Pibf1	Progesterone immunomodulatory binding factor 1	eBP‐1	Immune system process, regulation of interleukin production
ENSMUSG00000052430	1560	C104095	Bmpr1b	Bone morphogenetic protein receptor, type 1B	eBP‐1	Cytokine–cytokine receptor interaction pathway, TGF‐beta signaling pathway
ENSMUSG00000031309	3,690	C10961	RpS6ka3	Ribosomal protein S6 kinase polypeptide 3	eBP‐1	Toll‐like receptor 3 (TLR3) cascade pathway, TRAF6 mediated induction of NFkB and MAP kinases upon TLR7/8 or 9 activation pathway, Toll‐like receptor 2 (TLR2) cascade pathway
ENSMUSG00000020516	18,252	C147558	RpS6kb1	Ribosomal protein S6 kinase, polypeptide 1	eBP‐1	TGF‐beta signaling pathway, Fc gamma R‐mediated phagocytosis pathway

**Figure 6:**
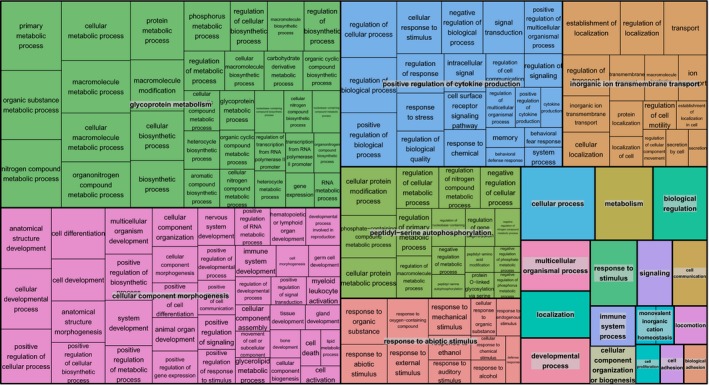
TreeMap view of REVIGO Biological Process analyses. Each rectangle represents a single cluster; the clusters are grouped into “superclusters” of related terms, represented with different colors. The size of the rectangles reflects the frequency of the GO term in the set of outliers included in this analysis

Among the gene‐associated GO terms, 445 had corrected *p*‐values < 0.05. Out of these, 341 belonged to the category “Biological Process.” REVIGO analysis formed 22 clusters for the category Biological Process (Figure 6), one of the three most represented one corresponding to the “positive regulation of cytokine production.”

For the 108 outliers associated to mouse genes, 125 Reactome/KEGG/INOH pathways were identified, out of which 13 were significantly enriched (corrected *p*‐values <0.05; Table 4). Three of them were directly linked to immune pathways (Toll‐like receptor cascades, JAK–STAT pathway and regulation, IL‐7 signaling) and one of them (VEGF signaling pathway) is known to be activated during hantavirus infection (Hepojoki, Vaheri, & Strandin, 2014).

## 4 DISCUSSION

The application of high‐throughput sequencing and genotyping technologies in zoonotic studies has been limited, so far, to the characterization of newly emerging pathogens (Yang, Yang, Zhou, & Zhao, 2008). The analysis of gene interactions that govern reservoir responses to pathogens still remains mostly restricted to laboratory models and major diseases (e.g., malaria vectors; White et al., 2011). In the present study, we used paired‐end RAD sequencing to examine the genomic patterns of differentiation among six natural populations of *Myodes glareolus,* a rodent reservoir of *Puumala* virus, the agent of a mild hemorrhagic fever with renal syndrome in humans. This study complements recent investigations on bank vole physiology (Konczal et al., 2016; Konczal, Babik, Radwan, Sadowska, & Koteja, 2015), gene evolution (Migalska, Sebastian, Konczal, Kotlik, & Radwan, 2017) and geographic expansion (White, Perkins, Heckel, & Searle, 2013). The RAD‐seq markers described here will further be available to study genetic diversity, population structure and adaptation in bank voles.

### 4.1 Genomic differentiation and limits of the sampling design

The phylogeography of bank voles is characterized by a bidirectional colonization of Sweden, resulting in a contact zone between two genetically differentiated mitochondrial lineages from southern and northeastern Sweden, respectively (Jaarola, Tegelstrom, & Fredga, 1999; Tegelstrom & Jaarola, 1998). The strong differentiation between these two lineages may be the consequence of hybridization between *M. glareolus* in northern Sweden and a closely related species, the red‐backed vole *Myodes rutilus *(Tegelstrom, 1987). Interestingly, Puumala viruses circulate on both sides of this contact zone, with distinct genetic variants in the northern and the southern bank vole populations, which suggests that a contact zone may also exist between genetically differentiated PUUV lineages (Hörling et al., 1996). Our sampling along a latitudinal transect ranging from PUUV‐endemic (northern Sweden) to non endemic (southern Sweden) areas is therefore characterized by a strong genetic structure, which overlaps ecological gradients as well as PUUV distribution. The strongest level of differentiation is observed between the northernmost population (Hörnefors) and the southern ones (pairwise *F*_ST_ comprised between 0.248 and 0.361), in line with the strong pattern of isolation by distance that we found.

The first consequence of our sampling strategy is that the outstanding differentiation of outliers may not result from local adaptation but from genetic incompatibilities between different backgrounds, that is, from combinations of alleles at different loci involved in negative epistatic interactions. Such endogenous genetic barriers may result in tension zones, the location of which is initially stochastic but tends to overlap with exogenous ecological barriers ("coupling effect"; see Bierne, Welch, Loire, Bonhomme, & David, 2011). It is nearly impossible to disentangle locally adapted genes from genetic incompatibilities in our case, but replicating this work in the future along other PUUV‐endemic–non endemic transects could help identifying loci commonly evolving in response to PUUV in bank vole populations, in a consistent way.

The second consequence of our sampling strategy is a suboptimal statistical power to detect local adaptation. Genome‐scan methods are often based on theoretical assumptions (e.g., equilibrium between evolutionary forces, island model of migration) that are rarely completely met in natural conditions. Moreover, spatial autocorrelations in allele frequencies due to isolation by distance can result in spurious correlations between such frequencies and environmental variables (Lotterhos & Whitlock, 2015; Meirmans, 2012). This may result in a larger number of false positive detected with SelEstim (which assumes an island model of population structure) as compared to BayPass, and therefore a low number of common outliers between SelEstim and BayPass. These limitations urged us to analyze genetic–environment associations, while controlling for population structure, using BayPass. However, this method may also have low statistical power with our sampling design due to the strong correlation between the main axis of population genetic differentiation (latitudinal pattern of isolation by distance) and the main axis of environmental variation (including variations in PUUV prevalence). Unfortunately, it was impossible for us to study pairs of nearby populations, as recommended by Lotterhos and Whitlock (2015), because the fine limit between geographic areas where PUUV prevalence is high versus low is barely known. We therefore had to consider a large sampling scale for bank vole populations, which led to highly genetically structured samples. Furthermore, the correlation of different variables along the main axis of environmental variation may cause the identification of outliers that are not involved in the adaptation to PUUV infections, but rather respond to other selective pressures, the distribution of which are correlated with NE distribution in Sweden.

Lastly, our sampling strategy consisted in the collection of samples during a single time step for each locality, although bank vole populations are known to exhibit strong multiannual density fluctuations, in particular in Fennoscandia (Henttonen, McGuire, & Hansson, 1985). A recent study provided evidence for temporal variations in both the direction and the efficiency of selection (Dubois, Galan, et al., 2017). Our results therefore need to be confirmed by analyzing temporal replicates within and between multiannual cycles of bank vole density in Sweden.

### 4.2 Methodological considerations

RAD‐seq has become an increasingly common genome‐scan approach this last decade, although several difficulties regarding the identification of genes of functional significance with regard to population divergence and local adaptation have been pointed out (Lowry et al., 2017). First, uneven contribution of individual DNAs to the pools may result in biased estimates of allele frequency (Rode et al., 2018). As recommended by Guo, Li, and Merila (2016) and Andrews, Good, Miller, Luikart, and Hohenlohe (2016), we minimized this bias by defining large sample sizes per pool and by replicating libraries for each population. Furthermore we only used methods that have been specifically designed to handle Pool‐seq data 2018, Vitalis et al., 2014, Gautier, 2015).

Second, the RAD‐seq approach enables to sample a limited fraction of *M. glareolus* genome (ca. 35 Mbp, *i.e.,* about 1%) so that potentially important adaptive SNPs might have been missed (Lowry et al., 2017). This limitation was reinforced by the lack of genomic resources for bank voles with genome annotations available that prevent us from identifying a large part of outlier SNPs. Only 44.4% of the outlier contigs blasted to genes in *M. musculus* genome, and 21% belonged to protein coding regions and could therefore be annotated. Altogether, these methodological limitations may explain why this population genomic approach could hardly confirm previously identified candidate genes, involved in the bank vole susceptibility to PUUV (Charbonnel et al., 2014).

### 4.3 Genes and pathways potentially involved in adaptation

Because of the limits listed above, the results from the genome scans have to be considered cautiously. The combination of three statistical analyses based on population differentiation and ecological associations to detect outlier loci aimed at reducing the rate of false positives and at enhancing our chances to detect genuine signatures of selection (François, Martins, Caye, & Schoville, 2016). As such, our study provides preliminary insights into potential immune pathways that could be involved in *M. glareolus* interactions with PUUV. Such information is important, since alternative approaches to tackle this question (e.g., PUUV experimental infections or long‐term population monitoring) are challenging.

We have found 14 genes associated with immunity, among the 95 outliers for which we obtained GO annotations. This proportion of immune‐related genes among our set of outliers is higher than what is expected from other genomes (e.g., 7% of immune‐related genes in the human genome, 11% in the house mouse genome). However, this comparison has to be taken cautiously, as the number of immune‐related genes is known to be highly variable across species (see, e.g., Emes, Eitan, Winter, & Ponting, 2003) and we do not have this information for the bank vole.

More precisely, we found evidence of association with PUUV infection for a small set of outlier loci (547 SNPs belonging to 468 contigs among 70,699 examined), showing high levels of genetic differentiation consistent with divergent selection acting along our latitudinal transect in Sweden. A significant fraction of these SNPS were related to immunity and responses to hantavirus infections. In particular, one third of the enriched biological processes associated with these SNPs corresponded to immune processes and “positive regulation of cytokine production” was one of the three most represented enriched pathways. These results strengthen the hypothesis that infectious pathogens are among the strongest selective forces acting in natural populations (e.g., Fumagalli et al., 2011, Karlsson, Kwiatkowski, & Sabeti, 2014, Vatsiou, Bazin, & Gaggiotti, 2016). Many of the immune‐related genes showing footprints of positive selection that we identified here were detected using ecological associations with a synthetic variable describing latitudinal variations in climatic, forest features and NE cases in humans. Although these associations do not reflect causality, they might be biologically meaningful with respect to environmental factors and *M. glareolus* microbiome, including PUUV, that influence bank vole responses to parasitism and genetic polymorphism. Further analyses and experiments are required to confirm and better interpret this result.

More specifically, the gene enrichment analyses emphasized potentially important receptor and signaling pathways known to be involved during hantavirus infections (Gavrilovskaya, Brown, Ginsberg, & Mackow, 1999; Gavrilovskaya, Gorbunova, Mackow, & Mackow, 2008; Handke, Oelschlegel, Franke, Kruger, & Rang, 2009). It included Toll‐like receptors, the inflammatory signaling pathway Janus kinase (JAK)–signal transducer and activator of transcription (STAT), the vascular endothelial growth factor (VEGF) and the interleukin‐7 (IL‐7) signaling pathways.

Toll‐like receptors are known to be potential pattern recognition receptors (PRR) for hantaviruses, being involved in early recognition of infections (Handke et al., 2009; Jiang et al., 2008). Their stimulation activates downstream signaling immune cascades with production of pro‐inflammatory cytokines and interferons (Akira, Uematsu, & Takeuchi, 2006), which are crucial for inducing a variety of innate antiviral effector mechanisms (Kawai & Akira, 2005). Interestingly, one of the candidate genes exhibiting signature of positive selection encodes for TLR7, a receptor recognizing single‐stranded RNA virus such as hantaviruses. *Tlr* genetic polymorphism could affect the recognition of these viruses, and hence the activation of immune cascades, with consequences in terms of virus replication (e.g., for Tlr7 gene expression between sexes; see Klein et al., 2004).

Interferons produced following TLR binding to viruses activate the JAK–STAT signaling pathway, which is essential in directing the immune response to virus infection. Moreover, hantaviral proteins have been shown to suppress the activation of this pathway (Levine et al., 2010; Schountz & Prescott, 2014), which is fatal in humans (Khaiboullina et al., 2017). Besides, VEGF is a cytokine that enhances endothelial cell permeability and shows elevated levels in hantavirus‐infected humans, suggesting that it could be involved in hantavirus pathogenesis (Gavrilovskaya et al., 2008; Gavrilovskaya, Gorbunova, Koster, & Mackow, 2012). In reservoirs, hantaviruses persist without exhibiting any sign of immune pathology and they evade immune responses to establish persistence (Easterbrook & Klein, 2008). Assessing the impact of genetic polymorphisms associated with JAK–STAT and VEGF pathways on bank vole immune responses to PUUV infections, and on PUUV persistence in its reservoir, could open new perspectives of research to better understand variations in tolerance to hantavirus infections.

Interleukin‐7 is a cytokine involved in the adaptive immune system and in particular in the development of B cells, T cells, and regulatory T cells. Its contribution to the response to hantavirus infections has only been scarcely shown in humans, with an upregulation observed during non fatal human infections compared to fatal cases (Khaiboullina et al., 2017). Again, its role in rodent/hantavirus interactions remains to be explored, especially with regard to its potential influence on regulatory T‐cell maturation (Easterbrook, Zink, & Klein, 2007), and in turn on rodent tolerance to hantavirus. There could be adaptive processes associated with these cells that would lead to reduced immunopathologies while resulting in the persistence of hantaviruses in infected rodent reservoir hosts (Schountz et al., 2007).

Altogether, further experimental approaches are required to validate the biological role of these genomic polymorphisms on bank vole tolerance to PUUV infections. Such studies would enable to test the hypothesis that this immunogenetic variability participates in shaping the contrasted PUUV epidemiological situations observed in natural populations of bank voles from northern and southern Sweden.

## 5 CONCLUSIONS

Using a pool RAD‐seq approach and a combination of statistical methods to detect loci with outstanding genetic differentiation and/or associated with environmental variables of interests, we have identified a list of putative loci that are worthy of further experimental and functional studies to better understand *Myodes glareolus*/PUUV interactions, and potentially NE epidemiology. These results are of main importance because our previous knowledge of the factors driving immuno‐heterogeneity and tolerance among reservoirs of hantaviruses was inferred from the medical literature or from results based on laboratory models. In the future, they could enable to better assess the risk of NE emergence by including reservoir immunogenetics in ecological niche modeling. They could also have important implications for medical purposes, by improving our understanding of the immune and metabolic pathways driving hantavirus pathogenesis in humans and non reservoirs.

## CONFLICT OF INTEREST

The authors declare that they have no competing interests.

## AUTHOR CONTRIBUTIONS

A.R., M.Gal., and N.C. designed the research. G.O. performed field sampling. A.R., M.Gal., and K.G. performed the laboratory work. N.C., A.R., B.G., R.V., and M.Gau. helped with bioinformatics and statistical analyses. C.Z. and S.VW. provided the environmental data. N.C. wrote the first draft of the manuscript, and all authors contributed substantially to revisions.

## DATA ACCESSIBILITY

DNA sequences of candidate genes are accessible in GenBank (see accession numbers in Supporting Information Table S1).

The Illumina RAD‐tag sequence dataset supporting the conclusions of this article will be available in the Dryad Digital Repository.

Our final SNP dataset is available as a supplementary file (Supporting Information Table S5).

## ETHICAL APPROVAL

Permission to trap voles was obtained from the Swedish Environmental Protection Agency (SEPA; latest permission: Dnr 412‐4009‐10) and from the Animal Ethics Committee in Umeå (latest permission: Dnr A‐61‐11).
