# Supplementary material for: Preliminary insights into the genetics of bank vole tolerance to Puumala hantavirus in Sweden
Source: Ecol Evol. 2018 Oct 26;8(22):11273–92. doi: 10.1002/ece3.4603 (PMC6262921; doi:10.1002/ece3.4603)
Supplement: Supplementary file 1 [file ECE3-8-11273-s001.docx]

**Figure S1:** Venn diagrams showing outliers that were unique to, and common among the three statistical methods (SelEstim, BayPass, BayPass with genetic-environment associations). The three graphs respectively represent results obtained when considering the thresholds based on a) 99.90%, b) 99.95% and c) 99.99 % quantiles calculated using calibration procedures or simulations.

a)


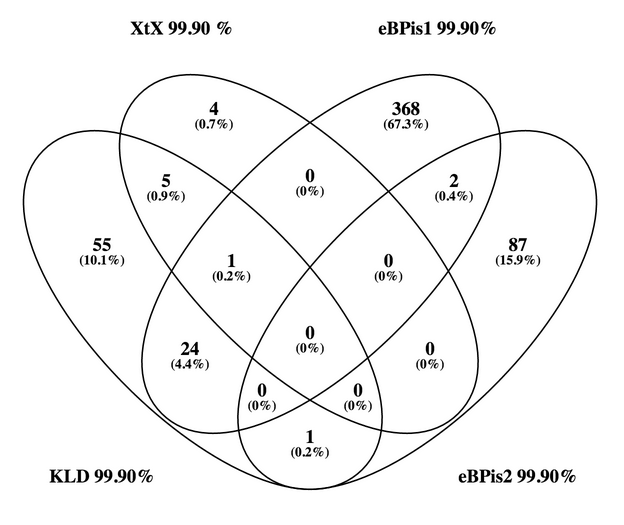


b)


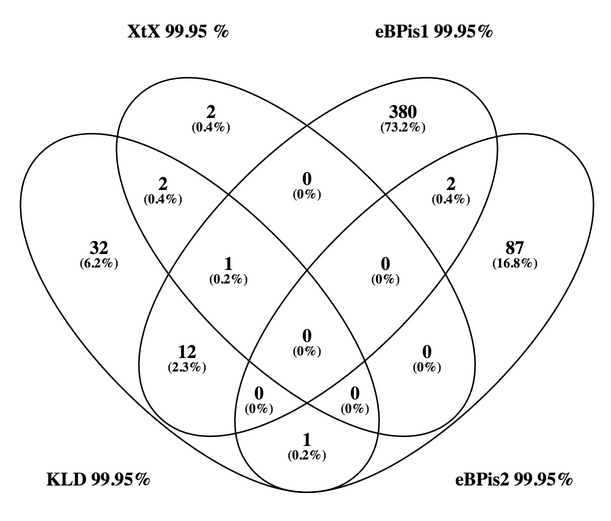


c)


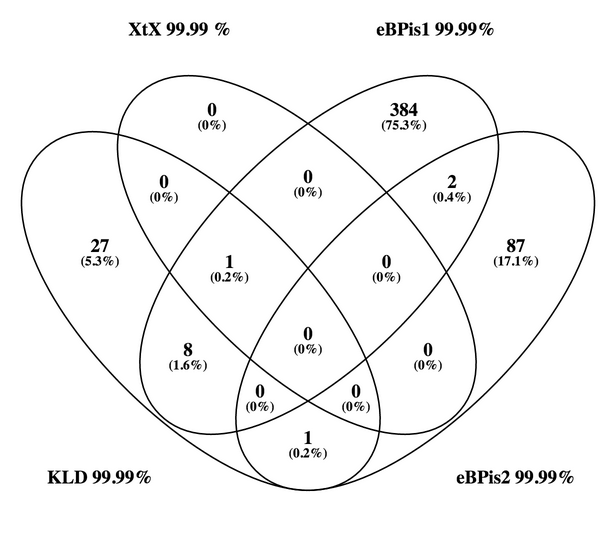


**Table S1:** Summary of the eight SNPs detected within candidate gene sequences.

| Gene | SNP^1^ | AA site^1^ | Codon Change | Syn/Nonsyn^2^ | Genbank |
| --- | --- | --- | --- | --- | --- |
| Myxovirus resistance (*Mx2*)  Exon 14 |  |  |  |  | KX463527 - KX463563 |
|  | 162 C/G | 54 |  | Syn |  |
| Toll-like receptor 4 (*Tlr4*)  Exon 3^3^ |  |  |  |  | KX463564 - KX463604 |
|  | 667 G/A | 222 |  | Non Syn |  |
|  | 776 G/A | 258 |  | Syn |  |
|  | 1146 C/T | 382 |  | Syn |  |
|  | 1662 A/G | 553 |  | Syn |  |
|  | 1687 C/T | 562 |  | Syn |  |
| Toll-like receptor 7 (*Tlr7*)  Exon 3^3^ |  |  |  |  | KX463605 - KX463616 |
|  | 2593 T/C | 865 | TTC (Phe) > CTC (Leu) | Non Syn |  |
| *Tnf* promoter |  | - | - | - | HM107872.1 |
|  | Tnf -296 | - | - | - |  |

^1^ SNP or amino-acid position relative to start of the brown rat (*Rattus norvegicus*) sequence. ^2^ Denotes whether the SNP is synonymous or non-synonymous. ^3^ We focused on the longest exons of *Tlr4* and *Tlr7* genes. We named them ‘exon 3’ by analogy with *Rattus* genome, which longest *Tlr4* and *Tlr7* exons are exon 3 (see Fornuskova et al., 2013). Note that the longest *Tlr* exon in *Mus* genome is exon 5.

FORNUSKOVA, A., VINKLER, M., GALAN, M., JOUSSELIN, E., PAGES, M., CERQUEIRA, F., CHARBONNEL, N., BRYJA, J. & COSSON, J. F. 2013. Molecular evolution of Toll-Like Receptors 4 and 7 genes in wild rodents (Murinae). *BMC Evolutionary Biology,* 13**,** 194.

**Table S2:** Detailed information about libraries, MIDs and Rad-sequencing results.

| Run name | Locality/library | MID sequence | Total number of reads | Number of RAD tag | Number of reads retained |
| --- | --- | --- | --- | --- | --- |
| RUN1 | Hörnefors1 | GATAA | 10776741 | 299067 | 10477674 |
| RUN1 | Hörnefors2 | GAAGTC | 9716829 | 297369 | 9419460 |
| RUN1 | Hörnefors3 | GGACG | 10101273 | 291697 | 9809576 |
| RUN1 | Hörnefors4 | GGTTCC | 9942519 | 225285 | 9717234 |
| RUN1 | Härnösand1 | GCCGC | 12307357 | 106479 | 12200878 |
| RUN1 | Härnösand2 | GCGAGC | 11589561 | 145259 | 11444302 |
| RUN1 | Härnösand3 | GTGTT | 11431209 | 238825 | 11192384 |
| RUN1 | Härnösand4 | GTTGGG | 11317782 | 321264 | 10996518 |
| RUN2 | Gnarp | AAGGG | 15268889 | 655480 | 13835114 |
| RUN2 | Gnarp | AAACAA | 12349131 | 343571 | 11335662 |
| RUN2 | Gnarp | ACAAT | 14843697 | 566747 | 13499346 |
| RUN2 | Gnarp | ACGTCA | 14977635 | 425546 | 13768060 |
| RUN2 | Njurunda | CGGAC | 16671261 | 442305 | 15345514 |
| RUN2 | Njurunda | CGTCAG | 13772673 | 400753 | 12663746 |
| RUN2 | Njurunda | CTAGA | 15597027 | 457525 | 14308742 |
| RUN2 | Njurunda | CTTATC | 14621044 | 419876 | 13416134 |
| RUN2 | Gimo/Tierp | GATAA | 14989477 | 400345 | 13801176 |
| RUN2 | Gimo/Tierp | GAAGTC | 14019001 | 414848 | 12864078 |
| RUN2 | Gimo/Tierp | GCCGC | 15165370 | 245998 | 14098772 |
| RUN2 | Gimo/Tierp | GCGAGC | 13607534 | 277785 | 12610244 |
| RUN2 | Enånger | TGTGT | 16069086 | 490006 | 14768096 |
| RUN2 | Enånger | TGCCCA | 14722127 | 366962 | 13584616 |
| RUN2 | Enånger | TTCAG | 14219669 | 560070 | 12936856 |
| RUN2 | Enånger | TTGGCC | 15318683 | 378616 | 14130040 |

**Table S3:** Pairwise Fst matrix between bank vole localities from Sweden.

|  | Enanger | Gnarp | Harnosand | Hornefors | Njurunda | Gimo/Tierp |
| --- | --- | --- | --- | --- | --- | --- |
| Enanger | NA | 0.09698287 | 0.2008016 | 0.3489692 | 0.11912346 | 0.1275186 |
| Gnarp | NA | NA | 0.1812733 | 0.3608364 | 0.09090006 | 0.1685516 |
| Harnosand | NA | NA | NA | 0.2486457 | 0.16815506 | 0.2405806 |
| Hornefors | NA | NA | NA | NA | 0.35719781 | 0.3397893 |
| Njurunda | NA | NA | NA | NA | NA | 0.1933696 |
| Tierp | NA | NA | NA | NA | NA | NA |
